# Museum spaces as psychological affordances: representations of immigration history and national identity

**DOI:** 10.3389/fpsyg.2015.00692

**Published:** 2015-05-27

**Authors:** Sahana Mukherjee, Phia S. Salter, Ludwin E. Molina

**Affiliations:** ^1^Department of Psychology, Gettysburg College, Gettysburg, PA, USA; ^2^Department of Psychology and Africana Studies, Texas A&M University, College Station, TX, USA; ^3^Department of Psychology, University of Kansas, Lawrence, KS, USA

**Keywords:** collective memory, identity, perception of racism, cultural psychology, assimilation

## Abstract

The present research draws upon a cultural psychological perspective to consider how psychological phenomena are grounded in socio-cultural contexts. Specifically, we examine the association between representations of history at Ellis Island Immigration Museum and identity-relevant concerns. Pilot study participants (*N* = 13) took a total of 114 photographs of exhibits that they considered as most important in the museum. Results indicate that a majority of the photographs reflected neutral themes (*n* = 81), followed by nation-glorifying images (*n* = 24), and then critical themes that highlight injustices and barriers faced by immigrants (*n* = 9). Study 1 examines whether there is a preference for glorifying images, and if that preference is related to cultural-assimilationist conceptions of national identity (i.e., defining American identity in dominant group standards). We exposed a new sample of participants (*N* = 119) to photographs reflecting all three themes. Results indicate that participants expressed greater liking for glorifying images, followed by neutral images, and critical images. National identity moderated within-subject variation in liking scores. Study 2 included 35 visitors who completed a survey before engaging with the museum or after their visit. Results indicate that participants who had completed their visit, compared to participants who had not entered the museum, reported (i) higher endorsement of cultural-assimilationist identity, and (ii) increased support for exclusive immigration policies. Study 3 exposed participants (*N* = 257) to glorifying, critical, or neutral images. Results indicate that participants who were exposed to glorifying images, especially those endorsing cultural-assimilationist identity, demonstrate decreased perception of current-day racial injustice, and increased ethnocentric enforcement bias. We discuss how engagement with privileged narratives may serve dominant group ends and reproduce systems of privilege.

## Introduction

In 2010, Arizona Governor Brewer signed two controversial bills into law. One bill (ArizonaHB 2281) enacted a ban on any courses that promoted “ethnic solidarity instead of treatment of pupils as individuals” or “resentment toward a race or class of people” among other things. Primarily focusing on Mexican-American, African-American, and Native-American history and literature courses, the ban embodies tendencies to associate mainstream education practices as “neutral” or “standard” while marking courses with race-conscious material as “ethnic,” “other,” and problematic. One of the most vocal proponents of the ban, Arizona State Schools Chief Tom Horne, claimed Mexican-American Studies “teach Latino students that they are oppressed by white people” (Cooper, 2010) when they should be “teaching these kids to be patriotic American citizens” (Ingram, 2014). As such, courses in Tucson’s largest school district were suspended because the race-conscious Mexican-American history textbooks were deemed non-compliant (Biggers, 2012). The other noteworthy bill signed during the same year (Arizona SB 1070) mandated stricter enforcement and policing of illegal immigration. The law required police officers (during routine stops, detentions, and/or arrests) to interrogate a person’s immigration status when there was “reasonable suspicion” that the person was unlawfully residing in the United States. Opponents in Arizona feared that the bill would sanction racial profiling and ultimately result in disproportionate harassment and discrimination against Hispanics, regardless of their citizenship status. Reflecting such concerns, Dr. Roberto Rodriguez, professor of Mexican American studies, stated that “the mood here is not anti-immigrant...the racial profiling has little to do with legalities; it is about the expressed targeting of red-brown Indigenous people” (Rodriguez, 2010).

The juxtaposition of the laws from the opening paragraph provides a contemporary example of how institutions participate both in reproducing desirable cultural narratives about the nation (e.g., excluding representations of cultural “others”) and sanctioning the consequences of not fitting into a particular national identity narrative (e.g., using race/ethnicity in judgments of reasonable suspicion). Taking this example as a point of departure, the present research applies a cultural psychological perspective to examine how cultural representations of a national past reflect and promote particular identity concerns (e.g., national identity, support for identity-relevant policies). By considering the extent to which “preferred” historical accounts reflect and serve dominant-group ends (e.g., White Americans in the U.S.), we also consider how representations of history (e.g., in museum spaces) can reproduce systems of privilege and disadvantage. Applied to the research topic, we conclude by discussing the systemic foundations of racial oppression.

## What is a Cultural Psychological Perspective?

While approaches vary (see Kim et al., 2012), the cultural psychology perspective that informs the current work considers psychological processes as forms of “mediated action” (Wertsch and Penuel, 1999). Informed by the works of Vygotsky (1978) and Bakhtin (1981), the concept of mediated action involves two elements: (1) the agent or the person who is doing the acting; and (2) the cultural tools present in the environment and used by the agent to accomplish a given action (Wertsch, 2002). For instance, consider the topic of memory. People can collectively remember a national past through engagement with cultural tools (e.g., museums and history curricula). The process of remembering is thus mediated through engagement with a particular tool present in the environment, and necessarily requires interaction with a given tool. From this perspective, memory is not limited to the biological underpinnings of brain architecture but also reflected in the social environment and reproduced through cultural practices and tools present in the environment. Similarly, consider the topic of national identity. A cultural psychological perspective suggests that rather than a natural connection to the nation, people construct an experience of national identity (i.e., identify with a nation and members belonging to a nation) based on an imagined community of other members who are distant in time and space. The process of imagination (of a national community) takes place through engagement with cultural tools (e.g., print media; Anderson, 1994). In this way, a cultural psychological approach is not limited to investigations of variation in psychological phenomena across cultural settings. Instead, the more fundamental point of this approach is to examine how apparently “natural” expressions of human psychology (e.g., national identity) require scaffolded engagement with cultural tools (e.g., cultural practices, language) in the environment.

**FIGURE 1:**
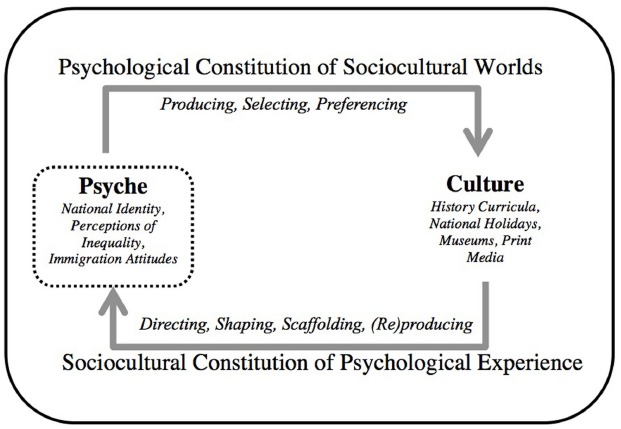
**Mutual constitution of culture and psyche**.

Furthermore, a cultural psychological perspective conceives of these various structures and patterns as cultural products that afford particular psychological experiences. That is, the products are not neutral in creation or subsequent impact. Instead, culture is shaped by people (i.e., product of action) and also shapes people (i.e., conditioning element for future action; Adams and Markus, 2004). In this way, culture and psyche make each other up in a bi-directional relationship of mutual constitution (Shweder, 1995). As shown in Figure 1, the top arrow refers to the *psychological constitution of sociocultural worlds*: the extent to which everyday ecologies are not “just natural” or do not develop out of “nowhere,” but are products of human action (Adams and Markus, 2004; Adams et al., 2010). From this perspective, cultural tools (e.g., museum spaces, history curricula) are products of human engagement and action, and may reflect the desires or beliefs of the people who created them. The bottom arrow in Figure 1 reflects the *sociocultural constitution of psychological experience*: the extent to which tendencies of human experience require engagement with the social context and thereby are not “just natural” or inborn (Adams and Markus, 2004; Adams et al., 2010). From this perspective, psychological experiences (e.g., conceptions of immigration history and national identity) require engagement with cultural tools present in any given context.

The present research applies a cultural psychological perspective to examine both aspects of the mutual constitution framework as it applies to the topic of national identity and representations of immigration history. In one direction, and corresponding to the top arrow of Figure 1 (*psychological constitution of sociocultural worlds*), we consider how conceptions of national identity influence people’s engagement with historical representations as well as understandings of present day accounts of injustices. In the other direction, and corresponding to the bottom arrow of Figure 1 (*sociocultural constitution of psychological experiences*), we consider how representations of history direct subsequent experiences in identity-relevant ways (i.e., national identity and support for policy).

### Representations of History: Tools for Regulating National Identity

A large body of work in the social sciences has examined the role of history in constructing and maintaining understandings of nationhood (Kohl, 1998; Reicher and Hopkins, 2001; Wertsch, 2002; Liu and Hilton, 2005). Because people do not have direct access to historical events, their knowledge of such events is mediated by engaging with textbooks (Loewen, 2007; Lackovic, 2011), museums (Rowe et al., 2002; Wertsch, 2007), memorials (Hirst and Manier, 2008), and commemorative practices (Kurtiş et al., 2010). Representations of history provide the scaffolding for conceptions of nationhood and other collective identities. People learn to attend to certain events in a national past, and learn to ignore or minimize other events, as they continuously engage with particular representations of history. Narratives that portray one’s group or nation in a positive light are canonized while the nation’s wrong-doings are silenced (e.g., Trouillot, 1995). Social psychological research, especially those that draw upon social identity theory and its related self categorization theory (Tajfel and Turner, 1986; Turner et al., 1987; Turner, 1989), suggests that people may be motivated to reinterpret or silence events that reflect poorly on their in-group, and which, by extension, reflect poorly on themselves (Branscombe et al., 1999). Accordingly, representations of history that are aligned with positive identities may be more likely to be (re)produced, compared to those are not aligned with positive identities. Moreover, these representations are in turn the products of prior action, and may also be associated with psychological characteristics of the original actors who produced the representations. Together, these ideas suggest that social representations of history serve as cultural tools in the production and maintenance of positive collective identities.

Within the mutual constitution framework, representations of history and the nation inextricably inform one another. In one direction, historical representations can influence identity-relevant experiences. For instance, reminders of an in-group’s past can have implications for how people feel about their group membership. Previous research indicates that reminders of the Holocaust—in particular the harmful actions committed by Germans—influence German participants to feel less positive about being German, compared to a control condition (Peetz et al., 2010). Historical accounts that highlight accounts of historical injustice and wrongdoing (vs. celebratory accounts of a nation) can influence beliefs about national identification. For instance, researchers have found that exposure to celebratory representations of American Thanksgiving that omitted any mention of historical instances of injustice (i.e., genocide) led to an increase in White American participants’ beliefs about national superiority, compared to representations that presented more critical accounts of Thanksgiving and acknowledged genocide (Kurtiş et al., 2010). This suggests that highlighting certain aspects of a historical event can influence people’s beliefs about a national community. By influencing national beliefs, historical representations can also play a role in reproducing narratives of conflict within a nation as well as conflict between nations. In an analysis of textbooks in Jewish schools in Israel, from the mid-1950s to mid-1990s, Bar-Tal (1999) found that most textbooks presented negative stereotypes of Arabs. Bar-Tal suggests that such negative stereotypes can maintain an Anti-Arab discourse in Israel and may contribute toward discriminatory forms of action. Extending this line of work, AL-Haj’s (2005) analysis of the revised textbooks, introduced in schools post 1999, indicates no mention of Arab experiences, possibly resulting in a removal of Arab citizens from the imagination of the Israeli community.

Besides “removing” groups of people from a national community (e.g., by not mentioning Arab experiences in history textbooks), representations of history may also have implications for how people respond to past and present-day issues of injustice. Responses to issues of injustice can in turn influence the extent to which individuals support or oppose the allocation of resources aimed at making amends for historical grievances (Sibley et al., 2008). Salter (2010) found that exposure to historical representations that emphasize racial barriers faced by Black Americans led White American participants to perceive a greater influence of current-day racism in American society, and endorse greater support for anti-racism policies, compared to representations that emphasized celebratory achievements of particular individuals (i.e., mainstream representations). Conversely, historical representations that focused on celebratory achievements (vs. historical injustices) promoted White American participants to deny current day issues of racism, and indicate lower support for anti-racism policies. Together, these examples indicate how historical representations can influence people’s level of national identification as well as detection of current day accounts of racial injustice.

In the other direction, historical representations are reflective of identity-relevant concerns. People’s recollections and engagement with particular accounts of history are associated with their collective identity. For instance, Sahdra and Ross (2007) found that participants who strongly identified (vs. weakly identified) with their religious group recalled fewer instances in which their group perpetuated violence against another religious group. In a second study, the same researchers found that when prompted to strongly identify with their nation, Canadian participants recalled fewer incidents of historical violence in which Canada committed harm/violence against another group, compared to those who were prompted to dis-identify. Thus, people may remember their past in identity-favorable ways to avoid negative feelings associated with threats to their identity (e.g., experience of collective guilt; Branscombe and Miron, 2004; Wohl et al., 2006). This is especially likely for those who highly identify with their in-group. People who are high in collective identification, compared to low identifiers, may reduce the negative consequences of engaging with in-group transgressions by psychologically distancing themselves from them (Pennebaker and Banasik, 1997), by not acknowledging the negative impacts of in-group transgressions (Doosje et al., 1998), or by shifting their standards of justice so that in-group wrongdoing no longer produces negative feelings such as collective guilt (Miron et al., 2010).

Importantly, people’s preferences for various accounts of the historical past may reflect their concerns about maintaining a positive identity. For instance, Kurtiş et al. (2010) found that White American participants who score high (vs. low) on a measure of national glorification indicated a preference for celebratory representations of American Thanksgiving (no mention of genocide) compared to ones that highlighted historical injustice (e.g., genocide). Similarly, Salter (2010) considered how mainstream Black history representations (prevalent in majority-White schools in the U.S.) reflect the preferences of White Americans. Salter (2010) examined Black History month representations in majority-White and majority-Black schools in the U.S., and found that majority-White schools tended to have mainstream celebratory representations while majority-Black schools tended to have representations that illuminated barriers and historical injustices. When exposed to Black history representations—mainstream celebratory representations as well as those highlighting historical injustice—White American participants reported more positive affect, and indicated a greater preference for celebratory representations, compared to representations of historical barriers and injustice. Moreover, the abovementioned effects were most evident among participants who strongly identified as being White American (compared to low identifiers). In sum, the abovementioned examples suggest that preferences for cultural products are aligned with identity-relevant beliefs (e.g., nation glorifying beliefs) present in these representations.

## Present Research

The present work examines the bi-directional relationship between national identity and historical representations on immigration. Particularly, it focuses on the *content* of national identity. So far, we have discussed research that illustrate how people—especially those who highly identify with their in-group—reproduce historical narratives that glorify their in-group’s past rather than those that highlight historical injustices and wrongdoings. More recently, scholars have considered how the *content* or meaning of national identity may moderate the relationship between strength or *level* of identification and treatment toward out-group. For instance, Smeekes et al. (2012) found that Dutch participants who highly identified with the Netherlands were more supportive of Muslim immigration to the Netherlands when exposed to historical narratives that framed Dutch traditions as being open and tolerant toward diverse religious faith, compared to exposure to narratives that emphasized the Christian history of the nation. Similarly, the positive relationship between nationalism—belief in national superiority—and support for biased treatment of immigrants was most evident for those participants who strongly endorsed an assimilationist understanding of American identity—the belief that to be truly American one must assimilate to dominant identity standards (Mukherjee et al., 2012). The present research examines a particular conception of national identity—assimilationist national identity—and applies it to the topic of immigration history in the U.S.

In one direction, we consider the extent to which historical representations on immigration are products of human action and reflect national identity concerns. In the other direction, historical representations are not inert end products, but direct experiences toward particular ends (e.g., impact national identity and support for immigration-relevant policies). More specifically, we consider the extent to which representations of American immigration history reflect conceptions of American national identity that serve dominant-group ends.

### Immigration Concerns and National Identity: Becoming a “true” American

The issue of immigration has been a topic of much discussion in the U.S. The last 5 years has seen many proposed policy changes, some of which focus on restricting movement of immigrants, possibly targeting those of Hispanic origin (e.g., SB 1070). Public opposition toward immigrants from Mexico and Latin America has often been widespread in the recent years (Pérez et al., 2008; Dovidio et al., 2010).

Without minimizing the role of economic concerns in opposition to immigration (see Valentino et al. (2013) for a review of economic explanations for opposition to immigration), several scholars have illuminated the role of symbolic concerns in understanding issues of immigration. More specifically, scholars have considered how immigrants especially those of Hispanic descent, and constituting the ethnic minority population, may pose a symbolic threat to Anglicized conceptions of American identity (Kinder and Sears, 1981; Zarate et al., 2004; Mukherjee et al., 2013; Yogeeswaran and Dasgupta, 2014). Theory and research demonstrate a conflation between U.S. national identity and White racial identity, and endorsement of this race-based national prototype—i.e., American is White—is associated with negative evaluations of ethnic minorities (Sidanius and Petrocik, 2001; Cheryan and Monin, 2005; Devos and Banaji, 2005; Devos et al., 2010; Yogeeswaran and Dasgupta, 2010; Huynh et al., 2014). White Americans consider ethnic minorities as less American especially when ethnic minorities fail to conform to dominant identity standards, and emphasize their allegiance toward their ethnic heritage, thereby threatening Anglicized conceptions of national identity (Yogeeswaran et al., 2012). Moreover, many Americans perceive Latin Americans as less “American” and question the legality of their presence in the nation (Dovidio et al., 2010). The above line of work suggests that White Americans may support tough stances on immigration to restrict the movement of racial and cultural others to protect against symbolic threats to dominant, Anglocentric constructions of American identity (Mukherjee et al., 2013). Simultaneously, White Americans may be less supportive of tough stances on immigration that restrict the mobility of people who do not threaten dominant constructs of American identity as White.

Support for the role of symbolic threat in tough stances on immigration comes from our previous research examining the identity correlates of ethnocentric bias in immigration law enforcement. For instance, endorsement of cultural-assimilationist conceptions of identity—the belief that to be “truly” American, one must conform to dominant American values (e.g., speak English)—is associated with punishing law-breaking immigrants but not law-breaking American employers who exploit immigrants (Mukherjee et al., 2012); and punishing law-breaking Mexican immigrants but not law-breaking Canadian immigrants (Mukherjee et al., 2013). This line of work suggests that anti-immigrant sentiments—especially those associated with assimilationist identity conceptions—may be linked with symbolic concerns about national identity, regardless of citizenship status.

Finally, White Americans perceive targets as more American when they conform to Anglo-centric norms (e.g., listen to American rock music, speak English with an “American” accent) compared to those who do not conform (e.g., listen to Mexican Ranchera music or Irish music; speak English with a “Spanish” accent), and perceptions of American-ness mediate participants’ judgments of law enforcement actions. That is, participants consider tough treatment of target (e.g., handcuff the target and detain target for being reasonably suspicious) as justified and fair when the target does not conform to Anglo-centric norms, compared to when the target conforms to dominant norms (Mukherjee et al., 2015). In sum, the above research examples suggest that an Anglicized conceptualization of American identity (i.e., cultural-assimilationist conception of identity) plays a significant role in privileging those who meet identity standards, and disadvantaging those who do not meet identity standards. Moreover, this conception of identity is not equally associated with anti-immigrant sentiments *per se*. Instead, it is associated with negative evaluations of those—citizens and immigrants—who do not conform to Anglicized standards.

### Emergence of Identity: A Cultural Psychological Analysis

How do such identity concerns develop and emerge? We draw upon a mutual constitution framework to consider the sociocultural grounding of identity concerns. Specifically, we examine the extent to which conceptions of cultural-assimilationist identity emerge through interactions with historical representations in an immigration museum. We also consider how identity concerns predict preferences for particular historical representations, and regulate people’s experiences with a cultural context (e.g., museum space). Scholars have noted how the history that people encounter in museum spaces, are often similar to what they may have experienced in their formal history education (e.g., in secondary schools; Barton, 2001). Thus, museums can serve as tools of history education and communicate institutional or official historical narratives (e.g., what *should* be remembered; see Rowe et al., 2002). Exposure to selective historical narratives may in turn inform visitors’ understanding of citizenship (e.g., what it means to be a true American). On the other hand, visitors may selectively engage with historical narratives (e.g., visit a particular exhibit and not visit others) and therefore shape their educational experience at the museum. This selective engagement may also be associated with their pre-existing conceptions of citizenship.

The present research utilizes a multi-method approach to examine the bi-directional relationship between conceptions of national identity and representations of immigration history present at the Ellis Island Immigration museum. In the pilot and Study 1, we consider whether participants are drawn toward particular representations of immigration history: those that glorify the nation and silence experiences of the marginalized cultural “others” versus those that highlight historical injustice and barriers that immigrants, especially those from historically oppressed groups, experience. Moreover, we also consider whether this differential preference—glorifying over historical injustice—is most evident for those participants who endorse cultural-assimilationist conceptions of American identity. Study 2 considers the extent to which engaging with historical representations at the museum space shapes people’s conceptualization of American identity as well as their support for immigration-relevant policies. Finally, Study 3 examines whether exposure to particular types of representations (i.e., nation-glorifying vs. critical accounts of injustice vs. neutral representations) influences identity-relevant experiences (i.e., perception of present-day injustice and support for policies). In the general discussion we consider the extent to which nation-glorifying representations of history serve dominant-group ends. We also identify alternative constructions of history that reflect experiences of the marginalized and promote liberatory outcomes.

## Pilot Study

We conducted the initial pilot study in the field to understand what type of museum content visitors to the Ellis Island Immigration Museum at New York City would be most likely to regard as important or noteworthy. Ellis Island is a small island in New York Harbor, located near the Statue of Liberty. It was used as an entry point for approximately 12 million immigrants between 1892 and 1924. More than a 100 million living Americans can trace their roots to an individual who passed through this island. This island became a museum site in 1990 and commemorated “American Immigrant Heritage” (Desforges and Maddern, 2004).

## Materials and Method

### Participants

Participants included 13 visitors to the Ellis Island Immigration Museum (9 women; *M =* 42.09 years old, SD = 5.57) in New York City. Out of 13, 10 people identified as White/Caucasian, 1 person identified as Latino/Hispanic, and 2 people did not respond to this item. Participants included 5 U.S. Born, 6 Non-U.S. Born, and 2 no responses. All participants approached had a digital camera at their disposal.

### Procedure

The first and second authors approached 63 participants who were about to enter the museum, and asked them if they would be willing to volunteer for a study that required taking pictures inside of the museum. Thirteen agreed to participate and were asked to take 10 photos of what stood out to them in the museum space (e.g., particular exhibits, certain artifacts, architecture, patrons of the museum). The researchers assured the participants that their photos would not be tied to any identifying piece of information (e.g., name). After completing their visit, researchers digitally transferred the photos from the participants’ cameras to an electronic tablet for short-term storage. Participants also completed a short survey to indicate their demographic information (i.e., age, gender, ethnic/racial identity, and nationality).

### Coding Photographic Content

To analyze the content of participants’ photos, two coders, blind to study hypotheses, used binary coding (yes or no) to indicate whether each photo contained critical themes, glorifying themes, and neutral themes. Critical themes were those that coders considered as focusing on historical injustice and that may make Americans (in general) feel negative (e.g., exclusionary literacy tests, forced migrations, hostility directed toward immigrant groups). Glorifying themes were those that coders considered as focusing on positive and glorifying aspects of history and that may make people in general feel proud of the U.S. (e.g., flag/patriotism, assimilation). Finally, neutral themes were those that coders considered ambiguous and that may not make people feel either positive or negative about being American (e.g., journey, museum neutral, personal/self story). Discrepancies in coding were resolved by a third independent coder. In general, coders had high levels of consensus (κ = 0.88) on the way they coded the themes within each photograph.

## Results and Discussion

The highest frequency count of photos fell into the thematic category of “neutral” (*n* = 81). The second highest frequency count was for photos that were coded as “glorifying” (*n* = 24) and the lowest frequency count was for photos that were categorized as “critical” (*n* = 9). On average, neutral themes (*M* = 0.40, SD = 0.92), were more common than glorifying and critical themes (*M* = –0.72, SD = 0.45), *F*(1, 113) = 77.97, *p* < 0.001, ${\text{η}}_{p}^{2}$ = 0.41. Glorifying themes (*M* = –0.60, SD = 0.81) were more common (or less uncommon) than critical themes (*M* = –0.84, SD = 0.54), *F*(1, 113) = 6.41, *p* = 0.013, ${\text{η}}_{p}^{2}$ = 0.05.

Out of 114 photographs, U.S. citizens took 90 photos and non-U.S. citizens took 18 photos. Participants who did not indicate their citizenship status took the remaining 6 photos^1^. Out of the 90 photos taken by U.S. citizens, 71.1 % was neutral (*f* = 64), 20 % was glorifying (*f* = 18), and 8.9 % was critical (*f* = 8). Out of the 18 photos taken by non-citizens, 77.8 % was neutral (*f* = 14), 22.2 % was glorifying (*f* = 4), and 0 % was critical. It is possible that participants, regardless of citizenship status, tended to take more glorifying photos, compared to critical photos. Given the small sample size, it is difficult to ascertain the generalizability of this result. Nonetheless, this pattern suggests an interesting avenue for future research.

These results provide partial support for the hypothesis regarding a nation-glorifying bias in engagement with representations of history. Participants took a significantly greater number of photographs of nation-glorifying representations compared to representations that highlighted historical injustices and barriers. However, the precise character of these differences remains unclear. Did participants fail to take many photographs of critical themes because they did not have knowledge about their existence in the museum (e.g., did not visit the exhibit because the museum’s audio guide did not direct them toward a particular exhibit) or because they disengaged with those themes and considered them irrelevant to understandings of immigration history? If participants were equally exposed to all three themes, would one see the same pattern?

## Study 1

Study 1 employs a larger sample and addresses the limitations of the pilot study by exposing participants to all three themes (i.e., glorifying, critical, and neutral) of photos and examining whether they demonstrate a preference for the various representations. Study 1 also examines how dominant group members’ preferences for various representations of history are associated with their conceptions of American identity.

### Participants

Participants were 119 undergraduates (66 women; *M* = 18.69 years old, SD = 1.02; all U.S. citizens) at a U.S. Southern university who indicated White/Caucasian race/ethnicity. Participants received partial course credit for completing the study.

### Procedure

After agreeing to participate in the study, participants viewed twelve photographs from the Ellis Island Immigration Museum within a Qualtrics survey. Four photographs focused on historical injustices associated with immigration (e.g., discrimination faced by East Asian immigrants) and constituted the *critical* condition. Four photographs glorified the nation (e.g., contrasted “peace” and “prosperity” prevalent in the U.S. with “hunger,” “ruin,” “famine,” “death,” and “desolation” prevalent in the immigrants’ nations of origin). These photographs constituted the *glorification* condition. Four photographs were neutral, meaning that their content did not criticize nor glorify the nation (e.g., discussed the various sea ports in the U.S. that acted as gateways for immigration). The majority of these photographs were selected from the pilot study (all photos in the *glorification* and *neutral* condition, and two photos in the *critical* condition). However, because of the low frequency of critical photographs taken by participants in the pilot study, the critical condition was supplemented with photos taken by the first and second authors from their visit to the museum.

We randomly assigned participants to view these photographs in one of two alternating conditions (i.e., one neutral, one glorifying, one critical, and repeat order OR one neutral, one critical, one glorifying, and repeat order). Participants rated each photograph on how much they liked it, how critical it was, and how patriotic it was. After completing the rating task, participants completed measures on national identification and demographics.

### Measures

#### Photograph Ratings

Participants responded to three evaluative questions using a 7-point scale ranging from 1 (*Not at all*) to 7 (*Very Much*). The questions were: *How much do you like this photo?; How patriotic is this photo*?; and *How critical of America is this photo*? We used the last two items as a manipulation check to assess whether glorifying photos were considered more patriotic than critical or neutral photos, and whether critical photos were considered more critical of American history compared to glorifying and neutral photos.

#### Cultural-assimilationist National Identity

The present work emphasizes how cultural tools (e.g., representations of history) are associated with content of national identity (i.e., what it means to be a “true” American) and identity-relevant action. Particularly, we were interested in what previous researchers have referred to as cultural-assimilationist constructions of national identity (Pehrson and Green, 2010; Mukherjee et al., 2012, 2013), which emphasize assimilation to dominant cultural ways of being (e.g., knowledge of English language in the U.S. context). To measure this construct, we adapted items from the ISSP Research Group (2009a,b) and followed previous research (Pehrson and Green, 2010; Mukherjee et al., 2012, 2013). Participants ranked 10 statements in response to what it means to be “truly” American. Two of these items tapped into cultural-assimilationist conceptions of identity (“to be able to speak English”; “have U.S. citizenship”; Mukherjee et al., 2012) while the remaining eight items served as filler items (e.g., to feel American). We subtracted raw ranking responses from 10 (i.e., the number of options). We created the cultural-assimilationist score by averaging scores for the two items that assessed this construct. Higher numbers on this score indicate higher ranking of this construct.

#### Demographics

Participants completed several demographic variables including political ideology and country of residence. Political ideology was rated on a scale ranging from 1 (*Very Liberal*) to 7 (*Very Conservative*).

## Results and Discussion

Recall that participants were exposed to all three themes (glorifying, critical, and neutral) in one of two alternating conditions (i.e., one neutral, one glorifying, followed by one critical OR one neutral, one critical, followed by one glorifying). The inclusion of item order condition did not influence significance of results, and therefore we did not include it as a covariate. The inclusion of political ideology as a covariate did modify results, such that, findings were less statistically significant. Moreover, the topic of immigration has been an issue of much debate amongst those with different political ideologies. Accordingly, we included political ideology as a covariate in our analyses.

### Evaluation of Historical Immigration Themes

We conducted repeated measures ANCOVAs to examine whether evaluation of representations of history differ as a function of their thematic content (glorifying, critical, and neutral).

#### Manipulation Check

Our manipulation check indicated that participants considered the glorifying themed-photos as more patriotic (*M* = 4.52, SD = 1.10), compared to both, critical themed and neutral themed photos (*M* = 2.85, SD = 0.94), *F*(1, 116) = 371.89, *p <* 0.001, ${\text{η}}_{p}^{2}$ = 0.76. Participants considered the critical themed photos (*M* = 4.13, SD = 1.24), as more critical of American history compared to both, glorifying and neutral themed photos (*M* = 2.91, SD = 1.34), *F*(1, 116) = 107.04, *p <* 0.001, ${\text{η}}_{p}^{2}$ = 0.48. Interestingly in this case, participants also considered the glorying themed photos (*M* = 3.17, SD = 1.47), more critical than neutral themed photos (*M* = 2.57, SD = 1.32), *F*(1, 116) = 54.67, *p* < 0.001, ${\text{η}}_{p}^{2}$ = 0.32. However, since both means are below the midpoint of the scale, we do not have strong evidence to suggest that participants considered these photos as “critical.”

#### Liking ratings

Results indicated that participants liked the glorifying photos the most (*M* = 4.08, SD = 1.05) followed by the neutral themed photos (*M* = 3.41, SD = 0.98), and the critical photos (*M* = 2.64, SD = 0.97), *F*(2, 116) = 130.46, *p* < 0.001, ${\text{η}}_{p}^{2}$ = 0.53. Contrast analysis indicate that participants liked the glorifying photos more than the critical photos, *F*(1, 116) = 60.33, *p* < 0.001, ${\text{η}}_{p}^{2}$ = 0.34, as well as liked the neutral photos more than the critical photos, *F*(1, 116) = 32.04, *p* < 0.001, ${\text{η}}_{p}^{2}$ = 0.22. There was no significant difference in liking ratings of glorifying and neutral photos, *F*(1, 116) = 2.11, *p* = 0.15, ${\text{η}}_{p}^{2}$ = 0.02. These results indicate that participants liked the critical photos the least. Taken together, results from the pilot and evaluation of liking ratings in Study 1 suggest that participants may have taken fewer photos reflecting critical-themes because they did not like them, whether or not they were aware of their existence in the museum.

The next set of analysis focused on the extent to which American identity predicted differential engagement with photo content.

#### Liking Ratings and Identity

**FIGURE 2:**
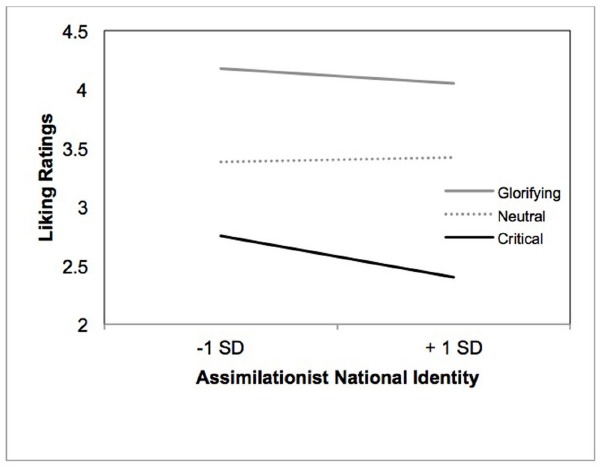
**Relationship between assimilationist national identity and liking ratings as a function of thematic content of the photos (Study 2)**.

We examined the extent to which endorsement of cultural-assimilationist conceptions of American identity predicted differential liking of photo content. Accordingly, we conducted a repeated measures analysis with identity as a continuous moderator of within-subject variation in liking ratings. As shown in Figure 2, results indicate a marginal interaction between identity and liking ratings, *F*(2, 115) = 2.44, *p* = 0.08, ${\text{η}}_{p}^{2}$ = 0.04. Simple slope analysis indicated that the more people endorsed a cultural-assimilationist conception of identity, the less they liked critical themed photos, *b* = –0.23, *t*(115) = –1.98, *p* = 0.05, ${\text{η}}_{p}^{2}$ = 0.03. There was no association between identity and liking ratings for neutral themed and glorifying themed photos, *p*s > 0.34. In sum, defining American identity in terms of dominant cultural values was negatively associated with liking critical-themed photos and unrelated to liking glorifying and neutral themed photos. The results so far (Pilot and Study 1) suggest that the relative absence of critical-themed photos in the pilot may be reflective of endorsement of a particular conception of identity, one that defines “true” Americans in terms of assimilation to dominant cultural views.

So far, results suggest that representations of history can reflect particular identity concerns: Photos that reflect historical injustices and highlight marginalized group experiences may not align with dominant conceptions of American identity. Accordingly, majority group members (e.g., White Americans) who endorse such dominant conceptions of identity may disengage from such representations (e.g., not consider them important to record during their visit to a museum because they dislike and disengaged from the content). Together, these results suggest that conceptions of American identity have several implications for engaging with historical as well as present day issues of injustice.

Does engagement with cultural products shape particular identity conceptions? Recall that a cultural psychological perspective proposes that identity does not emerge naturally (i.e., develop solely through psychological maturation). More specifically, the differences that emerge between those who endorse dominant conceptions of identity, and those who do not are not characteristic of stable, enduring traits inherent within individuals.

## Study 2

In Study 2, we consider how people’s conception of American identity is shaped by their engagement with their cultural worlds. Accordingly, Study 2 examines how engagement with representations of immigration history *influences* conceptions of identity and identity-relevant action. Moreover Study 2 included additional items on our identity measure, thereby providing a more comprehensive instantiation of our construct.

### Participants

Participants were 35 visitors to the Ellis Island Immigration Museum (18 women; *M* = 34.15 years old, SD = 18.46) in New York City, all of who indicated White/Caucasian race/ethnicity. Nineteen participants were on their way to the museum (i.e., “before” condition). Participants in the “before” condition included 13 U.S. citizens, 5 Non-U.S. citizens, and 1 individual who did not indicate their nationality. The remaining 16 participants had just completed their visit at the museum (i.e., “after” condition). These included 9 U.S. citizens, 2 non-U.S. citizens, and 5 individuals who did not report their nationality.

### Procedure

The first and second authors recruited visitors near the museum and asked them to complete a brief survey. Forty-three participants were approached either after they had just finished their visit to the museum (on Ellis Island) or as they were waiting in line to board the ferry that would take them to the museum (near Battery Park). Thirty-five participants agreed to participate in the study.

### Measures

#### Cultural-assimilationist National Identity

We used a similar ranking procedure as in Study 1. However, this time we included four additional items on cultural-assimilationist conceptions of identity. Participants ranked 10 statements in response to what it means to be “truly” American. Six of these statements tapped into cultural constructions of national identity (“be able to speak English” and “be Christian”). The remaining four items were filler items and focused on American identity but not related to cultural-assimilationist conception of identity (e.g., “be born in the U.S.). We subtracted raw ranking responses from 10 (i.e., the number of options). Higher numbers indicate that a participant placed greater importance on the associated identity characteristic. We created the cultural-assimilationist score by averaging scores for six items that assessed this construct. Higher numbers on this score indicate higher ranking of this construct.

#### Immigration Relevant Policies

We used three items to assess support for immigration relevant policies. Participants used a 7-point Likert scale (1 = *Not at all*, 7 = *Certainly*) to indicate their level of agreement to each item. The first immigration item focused on punishing undocumented immigrants: “States should have the right to question and detain anyone without proper identification who is suspected of being in the U.S. illegally.” The second item focused on a policy that promoted the use of bilingual education in schools (i.e., tapping into an inclusive stance on immigration): “States should support bilingual education programs in schools (practice of teaching non-English speaking students core subjects in their native language as they learn English).” The third item was also reflective of a more inclusive stance toward immigration: “The government should provide a “path of citizenship” for people who are in the U.S illegally.” We reverse-coded the item on detention and averaged the two items to create an immigration and bilingual education policy score. Higher scores on this measure indicated a more inclusive and favorable stance toward immigration relevant issues, α = 0.63.

#### Demographics

Participants completed several demographic variables including political ideology and country of residence (coded as 0 = U.S. and 1 = non-U.S.). Political ideology was rated on a scale ranging from 1 (*Very Liberal*) to 7 (*Very Conservative*).

## Results and Discussion

To examine how engagement with the museum influenced identity and policy support, we conducted one-way ANCOVAs with museum visit (before or after) as our between subject predictor. To maximize our sample, we included all participants, even those who were non-citizens of the U.S. To control for between-country variation, we used country of residence as a covariate in all analyses. While collecting the surveys from the participants, the first and second authors noted that several participants either failed to complete the measure on political ideology or verbally expressed their difficulty in completing that measure^2^. Participants who were non-residents of the U.S. indicated that their understandings of liberal and conservative were not aligned with American conceptions of the two constructs. Accordingly, we did not include this measure in our analysis.

### Cultural-assimilationist National Identity

Results^3^ indicated a significant difference such that participants who were surveyed prior to entering the museum indicated a lower endorsement of cultural-assimilationist constructions of identity (*M* = 4.89, SD = 1.46) compared to participants who had just completed their visit (*M* = 6.70, SD = 2.15), *F*(1, 21) = 5.65, *p* = 0.027, ${\text{η}}_{p}^{2}$ = 0.21. Stated differently, we found that participants who just completed their visit to the museum were more likely to define American identity in terms of assimilation to dominant cultural standards, compared to those who had not engaged with the contents of the museum.

### Immigration Relevant Policies

Results^4^ indicated a difference in policy support: Participants who were surveyed prior to entering the museum had more inclusive stances toward immigration policies (*M* = 5.20, SD = 1.25) compared to participants who had just gone through the museum (*M* = 4.15, SD = 1.35), *F*(1, 26) = 4.86, *p* = 0.036; ${\text{η}}_{p}^{2}$ = 0.16. Stated differently, we found that participants who just completed their visit to the museum, compared to those who were on the way to the museum, indicated increased support for detention of immigrants, and decreased support for bilingual educational policy as well as decreased support for a policy promoting a path to immigrant citizenship.

In sum, this study suggests that engaging with the museum content influences visitors’ understanding of the meaning of American identity (i.e., what it means to be a “true” American) as well as impacts their support for immigration-relevant policies. However, it is unclear whether this effect is because participants engaged with particular representations: For instance, is it reflective of the failure to engage with critical representations focusing on injustice, or is it reflective of an over-emphasis on nation-glorifying representations? Do prior conceptions of American identity influence the consequences of engaging with particular representations?

## Study 3

Study 3 addresses the questions raised in Study 2 by exposing participants to either glorifying, critical, or neutral images, and examining how differential exposure can influence how participants engage with current-day immigration issues (i.e., perception of injustice and support for immigration policies). Moreover, Study 3 also examines how people’s conceptions of American identity moderate the consequences of their engagement with particular historical representations.

### Participants

Participants were 257 undergraduate students (62.6% women; all U.S. citizens) at a U.S. Southern university. Participants received partial course credit for completing the study. Reported racial/ethnic background included: 64.6% European American/White, 18.3% Hispanic/Latina, 9.3% Biracial/Multiracial, 3.1% Asian American, 3.5% African American/Black, and 0.8% American Indian/Alaskan Native. Ages ranged from 18 to 28 (*M* = 18.80, SD = 1.13).

### Procedure

After agreeing to participate in the study, participants evaluated three photographs within a Qualtrics survey on a computer in a private cubicle. Participants were randomly assigned to view one of three conditions: *critical, neutral, or glorifying* images from the Ellis Island Museum. Participants rated each photograph on how much they liked it, how critical it was, and how patriotic it was. After completing the rating task, participants completed measures of cultural-assimilationist national identity, perception of racism, policy-support, and demographics.

### Measures

#### Cultural-assimilationist National Identity

Participants completed the same measure of identity as in Study 2.

#### Perception of Racism

Participants responded to seven items that assessed perceptions of racism in the context of immigration affairs (α = 0.78; see Adams et al., 2006). Participants used a 7-point scale (1 = *not at all due to racism*, 7 = *certainly due to racism*) to indicate the extent to which particular policies and state of affairs related to U.S. immigration was related to racism. Example items are *use of techniques such as racial profiling to identify and question people about their legal status*, *enacting stricter border security along the Mexican border*, and *using the term “alien” to refer to immigrants*.

#### Immigration Policy

We used four items to assess support for immigration policies similar to Arizona SB 1070. Participants used a 7-point Likert scale (1 = *Strongly Disagree*, 7 = *Strongly Agree*) to indicate their level of agreement to each item. As in Mukherjee et al. (2012), two of these items focused on policies that punished undocumented immigrants (“*States should have the right to question and detain anyone without proper identification who is suspected of being in the U.S. illegally*” and “*States should have the right to question people about their immigration status if they suspect they are unlawful residents of the nation*”). We averaged these two items to form an index of *immigrant-focused law enforcement* (α = 0.83). The remaining two items focused on punishing U.S. employers who exploited undocumented immigrants (“*Authorities should prosecute and punish Americans who exploit illegal immigrants for their labor or other services*” and “*Authorities should penalize*, *jail or otherwise punish American businesses that knowingly recruit and exploit undocumented immigrants*”). We computed the mean of these items to form an index of *employer focused law enforcement* (α = 0.69).

#### Political ideology

As in Studies 1 and 2, political ideology was rated on a scale ranging from 1 (*Very Liberal*) to 7 (*Very Conservative*).

## Results and Discussion

Table 1 presents the means and standard deviations of all measured variables.

**TABLE 1:** **Means and (Standard Deviations) of measures (Study 3)**.

	**Glorifying**	**Neutral**	**Critical**
Patriotic rating	4.47^a^ (1.26)	2.95^b^ (1.31)	2.20^c^ (1.22)
Critical rating	3.36^a+^ (1.39)	2.97^b^ (1.16)	4.60^c^ (1.40)
Liking rating	3.49^a^ (0.87)	3.40^a^ (1.09)	2.04^b^ (1.11)
National identity	4.75^a^ (0.67)	4.92^a^ (0.64)	4.97^a^ (0.75)
Perception of racism	4.12^a^ (1.38)	3.89^a^ (1.48)	4.14^a^ (1.31)
Policy: immigrant focused	5.04^a^ (1.67)	4.94^b^ (1.75)	4.61^c^ (1.73)
Policy: employer focused	4.58^a^ (1.47)	4.52^a^ (1.64)	4.49^a^ (1.57)

Standard deviations are in parenthesis. We report significant differences of pairwise comparisons, and different letter superscripts within rows indicate statistically significant (p < 0.05) differences; ^+^p < 0.1.

### Manipulation Check

We conducted 3 (photo theme: critical/neutral/glorifying) between-subjects ANCOVAs with political ideology as a covariate, to examine whether (i) participants considered glorifying themes as more patriotic, compared to critical and neutral, and (ii) participants considered critical photos as more critical of American history compared to glorifying and neutral photos.

#### Patriotic Rating

The omnibus ANCOVA was significant, *F*(2, 255) = 68.05, *p* < 0.001, ${\text{η}}_{p}^{2}$ = 0.35. Participants considered the glorifying themed-photos as more patriotic (*M* = 4.47, SD = 1.26) compared to the neutral photos (*M* = 2.95, SD = 1.31; *p* < 0.001), and the neutral photos more patriotic compared to the critical themed photos (*M* = 2.20, SD = 1.22; *p* < 0.001).

#### Critical Rating

The omnibus ANCOVA was significant, *F*(2, 255) = 37.39, *p* < 0.001, ${\text{η}}_{p}^{2}$ = 0.23. Participants considered the critical themed photos (*M* = 4.61, SD = 1.40), as more critical of American history compared to glorifying themed photos (*M* = 3.36, SD = 1.39; *p* < 0.001), and the glorifying themed photos as more critical than neutral themed photos (*M* = 2.97, SD = 1.16; *p* = 0.068). As in Study 1, the means for neutral and glorifying photos were both below the mid-point of the scale, thereby suggesting that participants did not consider these photos as “critical” *per se*.

### Liking Ratings

To test for differences in liking ratings between the various history themes, we conducted ANCOVA analysis with photo theme as the between subjects variable and political ideology as a covariate. The omnibus ANCOVA was significant, *F*(2, 255) = 53.50, *p* < 0.001, ${\text{η}}_{p}^{2}$ = 0.30. Pairwise comparisons indicated that participants liked the critical photos (*M* = 2.04, SD = 1.11) less than the glorifying themed photos (*M* = 3.49, SD = 0.87: *p* < 0.001) and less than the neutral themed photos (*M* = 3.40, SD = 1.09; *p* < 0.001). There was no difference in liking glorifying and neutral themed photos (*p* = 0.49).

## Regression Analysis

To investigate the effects of museum content and national identity content on perceptions of racism and policy support, we conducted two hierarchical regression analyses (Aiken and West, 1991). The effect of photo theme was decomposed using two orthogonal contrasts. The first contrast tested the hypothesized linear effect of photo exposure (critical = –1/neutral = 0/glorifying = 1). The second contrast tested the residual variance by comparing the neutral condition to the critical and glorifying conditions (critical = –1/neutral = 2/glorifying = –1). We entered the main effects—two contrasts and identity—on the first step, and two-way interactions between each contrast and identity on the second step. We mean centered cultural-assimilationist national identity before entering it in the multiple-regression models. We included political ideology as a covariate in all analyses^5^.

### Perception of Racism

**FIGURE 3:**
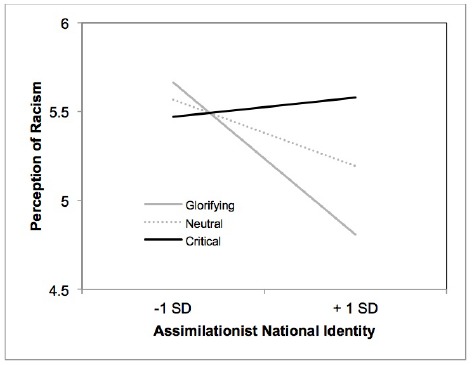
**Relationship between assimilationist national identity and perception of racism as a function of thematic content of the photos (Study 3)**.

Regression analysis indicated that there were no main effects of contrast 1, *b* = –0.09, *t*(249) = –0.82, *p* = 0.41, and contrast 2, *b* = –0.08, *t*(249) = –1.44, *p* = 0.15. There was a marginal effect of identity such that endorsement of cultural-assimilationist conception of identity was associated with lower levels of racism perception, *b* = –0.22, *t*(249) = –1.79, *p* = 0.07, ${\text{η}}_{p}^{2}$ = 0.01. That is, those who defined American identity in terms of dominant cultural values were less likely to perceive systemic acts of injustice in American society. Moreover, the higher order interaction between identity and the first contrast was also significant, *b* = –0.37, *t*(247) = –2.52, *p* = 0.01, ${\text{η}}_{p}^{2}$ = 0.03, but not the interaction between identity and the second contrast, *b* = 0.03, *t*(247) = 0.35, *p* = 0.73, thereby suggesting a linear moderating effect of photo theme on the link between identity and perception of racism. As depicted in Figure 3, simple slope analyses indicated that the negative relationship between defining American identity in cultural terms and racism perception was particularly evident among participants viewing the glorifying images from the Ellis Island Museum, *b* = –0.62, *t*(247) = –3.05, *p* = 0.003. Similarly, neutral images also decreased perceptions of racism among those participants defining American identity in terms of dominant cultural values, *b* = –0.27, *t*(247) = –2.15, *p* = 0.03. On the other hand, exposure to critical images, attenuated the negative relationship between assimilationist national identity and perception of racism, *b* = 0.08, *t*(247) = 0.42, *p* = 0.68.

In sum, results indicate that exposure to glorification and neutral images supported the negative relationship between assimilationist national identity and perception of racism. On the other hand, exposure to critical images attenuated the negative relationship between assimilationist national identity and perception of racism.

### Immigration Policy

We utilized two sets of policies: one that focused on tough treatment of undocumented immigrants and one that focused on tough treatment of American employers who exploit undocumented immigrants. To the extent that cultural-assimilationist conception of identity is associated with privileging those who meet dominant group standards (e.g., American employers), and disadvantaging those who do not meet these standards (e.g., undocumented immigrants), one can hypothesize that identity will impact within-subject variation in support for these types of policies. As in Mukherjee et al. (2012), we created a difference score by subtracting the *employer-focused* index from the *immigrant-focused* index. This difference score measure served as an index of ethnocentric enforcement bias and measured the extent to which participants supported the punishment of law-breaking immigrants over law-breaking American employers. To examine the extent to which national identity and conditional exposure influenced this bias, we conducted a hierarchical regression analysis as in earlier analysis.

**FIGURE 4:**
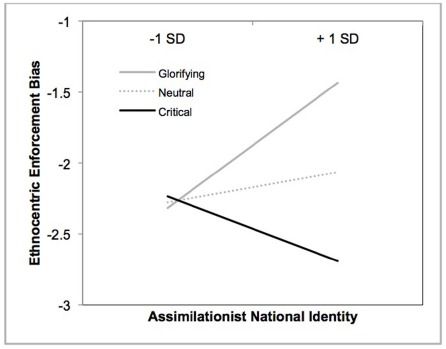
**Relationship between assimilationist national identity and ethnocentric enforcement bias as a function of thematic content of the photos (Study 3)**.

Regression analysis indicated that there were no main effects of identity, *b* = 0.13, *t*(249) = 0.72, *p* = 0.47, and contrast 2, *b* = 0.04, *t*(249) = 0.46, *p* = 0.65. There was a marginal effect of contrast 1, *b* = 0.28, *t*(249) = 1.82, *p* = 0.07, ${\text{η}}_{p}^{2}$ = 0.01, thereby suggesting a linear effect of photo theme on the ethnocentric enforcement bias. Moreover, there was a two-way interaction between contrast 1 and identity, *b* = 0.43, *t*(247) = 2.03, *p* = 0.04, ${\text{η}}_{p}^{2}$ = 0.02, and no significant interaction between contrast 2 and identity, *b* = 0.09, *t*(247) = 0.66, *p* = 0.51. As can be seen in Figure 4, simple slope analyses indicated that the positive relationship between defining American identity in terms of dominant standards and ethnocentric enforcement bias was most evident amongst participants who viewed glorifying images, *b* = 0.64, *t*(247) = 2.15, *p* = 0.03. There was no relationship between identity and ethnocentric enforcement bias for those who viewed neutral images, *b* = 0.15, *t*(247) = 0.84, *p* = 0.40, and those who viewed critical images, *b* = –0.33, *t*(247) = –1.19, *p* = 0.24.

In sum, results indicate that the effects of critical exposure trended in the opposite direction of the glorifying and critical directions. This suggests that exposure to critical images, compared to exposure to glorifying and neutral images, attenuated the positive relationship between assimilationist national identity and ethnocentric enforcement bias—the tendency to punish undocumented immigrants over American employers who exploit undocumented immigrants.

## General Discussion

The present work draws upon a diverse methodological approach—field research involving both quantitative and qualitative analysis, and experimental research—to examine how national identity concerns are aligned with representations of immigration history. In one direction (and associated with the *psychological constitution* direction of the mutual constitution framework), people’s preferences for historical representations are reflective of identity-concerns. In the other direction (and associated with the *sociocultural constitution* direction of the mutual constitution framework), historical representations promote identity-relevant experiences.

Evidence for the *psychological constitution* direction comes from the pilot study and Study 1. As part of the photo-task, participants in the pilot selected more nation-glorifying images compared to images focusing on historical injustice. When exposed to photographs reflecting neutral, nation-glorifying, and injustice themes, participants in Study 1, indicated a stronger preference for nation-glorifying themes, compared to neutral themes, followed by themes focusing on historical injustice. Alternatively stated, participants preferred the critical-themed images the least, compared to the neutral and glorifying themed-images. National identity moderated participants’ differential liking for historical representations. Defining American identity in terms of dominant cultural values was negatively associated with liking critical-themed photos and unrelated to liking glorifying and neutral themed photos. These results suggest that those who define identity in ways that fit with dominant group standards are less likely to engage with representations that focus on historical injustices. Repeated acts of preferential (dis)engagement may further reproduce a nation-glorifying bias in historical representations. For instance, after a visit to a museum, person A may discuss her experience with another individual, person B, and share her photographs with person B. If person A took more nation-glorifying representations (or less critical representations), then she is likely to influence person B’s knowledge of immigration history: person B may now be ignorant of events that focused on oppression and thus be unaware of the experiences of particular immigrant groups. Person A may also share her pictures on a social networking site and thereby influence a larger group’s understanding of immigration history. In sum, an emphasis on nation-glorifying representations may influence not just the visitor’s understanding of immigration history, but also, influence other people’s understandings of the past.

Evidence for the *sociocultural constitution* direction comes from Studies 2 and 3. We found that museum spaces direct people toward certain ends: In Study 2, participants who visited the museum were more likely to define American identity in terms of dominant group values (i.e., assimilationist identity) and indicate exclusive stances toward immigration issues (e.g., oppose bi-lingual education), compared to those who were on their way to the museum (i.e., waiting to take the ferry to the museum location). Study 3 results indicated that the negative relationship between American assimilationist identity and ethnocentric stances toward immigration (i.e., racism perception and policy bias) was true only for those participants who were exposed to glorifying images. Glorifying images thus promoted (i) the denial of racism and (ii) endorsement of ethnocentric enforcement bias especially amongst those participants with an identity profile (i.e., high cultural-assimilationist identity) conducive to anti-immigrant stances, or more specifically anti non-European/Anglo immigrant stances^6^. In contrast, results suggest that critical images may have served as an overriding influence and negated the negative effects of cultural-assimilationist national identity. In sum, these results suggest that the contents of museums are not just products of human activity, but they also shape psychological experiences in ways that may serve dominant group ends (e.g., resonate with dominant conceptions of identity).

Together, results across the pilot and three studies suggest that the presence and absence of particular historical representations may not emerge by accident. Instead, they may be reflective of specific identity concerns. Representations that are more consistent with dominant group identity concerns (i.e., aligned with cultural-assimilationist conception of identity) may be preferred and selected for considerations of immigration history (e.g., photographed and included in photo albums). Repeated acts of preferential selection may then influence the cultural rhetoric of immigration (e.g., concerns on what gets included/excluded in academic curricula). Results also suggest that historical representations direct people to define American identity in particular ways and influence identity-relevant experiences (e.g., racism perception). Conversely stated, identity conceptions do not emerge naturally but are products of engagement with one’s sociocultural context. Finally, exposure to particular historical representations can promote or override the ethnocentric tendencies of cultural-assimilationist conceptions of identity; in particular, consider the impact of nation glorifying themes versus themes reflecting historical wrongdoing on anti-immigration policy support. Together, results from all studies provide support for the bi-directional relationship between cultural context (i.e., historical representations) and psychological experience (i.e., national identity and identity-relevant experiences).

## Limitations and Future Directions

### Restricted Sample Sizes

One aim of the project was to consider the photographs taken by museum visitors and consider how a new group of participants would respond to the themes reflected in the photographs. Although participants provided the researchers with over 100 images to analyze, a limitation of the pilot study was that the photographic stimuli was derived from a limited number of participants. Accordingly, it is difficult to ascertain whether the lack of critical representations in photographs can be generalized to a larger sample. Moreover, the restricted sample size also made it difficult to ascertain the extent to which participants’ identity characteristics (e.g., the photographer’s conception of American identity) were associated with their selection decision (i.e., consideration of photographs with certain themes). Is it the case that people who endorse cultural-assimilationist conceptions of identity fail to take photographs reflecting critical themes? With a larger sample size, future research could consider the extent to which national identity or additional individual difference characteristics (e.g., conflict avoidance) are associated with selection decisions of the sample.

### Which Representations Make an Impact?

Study 3 results indicated that the negative relationship between identity and racism perception, and the positive relationship between identity and policy bias were most evident amongst those who viewed glorifying images. In contrast, results did not reveal statistically significant relationships between identity and these identity-relevant tendencies among those who viewed images that were critical of American history. How does one interpret these results? On one hand, this suggests that glorifying photos may already resonate with dominant-identity conceptions and therefore reproduce the negative relationship between cultural-assimilationist identity and ethnocentric stances toward immigrants. This implies that the “standard” conditions that maintain this association are nation-glorifying (or are not critical of national history) and therefore merely strengthen this association. Previous research has found a consistent association between this conception and ethnocentric stances toward immigrants (see Pehrson and Green, 2010; Mukherjee et al., 2012, 2013). To the extent that the “neutral” condition serves as a control, there is evidence for this initial interpretation. On the other hand, this does not rule out the interpretation that exposure to historical accounts of oppression attenuates the “natural” association between identity and ethnocentric tendencies, and therefore conducive to *countering* the reproduction of hierarchy-enhancing, dominant-group ends. There is some evidence to support this interpretation (see decreases in anti-immigrant policy bias in Table 1, Study 3), but the evidence is minimal. However, we find it noteworthy that despite being non-significant, the effects of critical exposure trended in the opposite direction of the glorifying and neutral conditions. This suggests to us the possibility that repeated or longer-term exposure to critical narratives would be necessary to fully reverse the existing negative relationship between identity and racism perception (and the positive relationship between identity and policy bias). Furthermore, it is also possible that the limited impact of critical-themed photos is due to the competing motives in response to the images. That is, participants may be experiencing both a motivation to utilize the photos as information (i.e., immigrants face numerous hardships, including ethnic exclusion) and a motivation to dismiss the photos because they negatively implicate the group (i.e., these accounts of ethnic exclusion do not align with my perception that the nation is moral). Alternatively, exposure to past accounts of wrongdoing may increase consciousness for some, but may also increase threat for others, especially amongst those who identify with the perpetrator category. Future research should investigate the conditions in which, and/or for whom, critical-themed representations lead to more positive social outcomes (e.g., increased racism perception).

## Conclusion

A key contribution of the present work is the application of a cultural psychological perspective toward the study of historical representations and identity. People rely upon historical representations (e.g., museums, history curricula) to learn about a nation’s past (Wineburg, 2001; Loewen, 2007). Moreover, they learn to attend to certain events (e.g., nation glorifying events) and learn not to attend to certain events (e.g., critical events focusing on injustice) as they continuously engage with such representations. Representations of the past are in turn regulated by preferential selection tendencies of prior actors (e.g., individuals who create museum space, design curricula). That is, historical representations in museum spaces do not emerge from nowhere. People design a museum and make decisions to include or exclude particular representations. The present work suggests that national identity influences people’s preferences for particular historical representations. Those who define American identity in terms of dominant-group values prefer nation-glorifying representations compared to critical representations that focus on historical injustices and barriers. These representations in turn serve as repositories of knowledge and influence subsequent identification and identity-relevant tendencies. Continuous engagement with particular representations of history present in museum spaces may further strengthen (or reduce) dominant conceptions of national identity. Moreover, particular representations (e.g., glorifying images) may be conducive for minimizing current issues of racial injustice and increasing biased support for law-enforcement policies. This suggests that historical representations or historical sites can direct future behavior and action of those who engage with these representations. An important implication of this form of analysis is that, in directing people toward certain ends (e.g., consistent with cultural-assimilationist conceptions of identity), a historical site may also lead people to ignore other ends (e.g., critical representations that reflect injustice and is inconsistent with dominant-group ends). If one narrative is privileged, another is silenced or ignored. Thus, museum sites may play a dynamic role in constructing “knowledge” and provide basis for revealing the epistemologies of ignorance (Mills, 2007). Historical representations can direct one to behave in certain ways *and* to not behave in other ways (e.g., promote ways of knowing that contribute toward the denial of injustice).

The present work also contributes to a cultural psychological approach to topics of injustice and oppression. Research on social inequalities has mainly focused on the role of individual stereotyping, prejudice, and discrimination (Adams et al., 2008). Such “standard” framings of injustice (e.g., racial injustice) can promote the construction of injustice as a problem of biased acts of prejudiced individuals, and minimize the systemic roots of injustice (Hopkins et al., 1997; Adams et al., 2008). We draw upon a cultural psychological perspective to consider the systemic factors that reproduce racial inequalities. Particularly, we consider how people can be immersed in an environment that is structured in a way to reflect and reproduce dominant-group interests. We also consider alternative arrangements that promote more inclusive ends. Our results indicate that historical representations that focus on national glorification are more prevalent in judgments of national history (i.e., considered more important to photograph and record). The reproduction of such representations lays the foundation for dominant-identity concerns (e.g., assimilation to dominant-group standards, denial of injustice). In contrast, alternative representations of history that reflect experiences of historically oppressed immigrant groups and highlight social injustice may be more aligned with the detection of present-day experiences of injustice, and provide bases for more inclusive and reparative action (e.g., supporting bi-lingual education; see Martín-Baró, 1994). Thus, museums can also serve as pedagogical tools that promote positive and inclusive outcomes.

In conclusion, a cultural psychological perspective emphasizes the mutually constitutive relationship between culturally patterned worlds and human psychological experience. On one hand, results suggest that museum spaces, like all sociocultural contexts, can privilege certain representations (e.g., those that emphasize national glorification) and those representations shape how we see the world. In this case, the immigration museum promotes conceptualizing what it means to be American as assimilating to dominant cultural values. On the other hand, how might some narratives find prominence in any given sociocultural context? These results also suggest that the presence or absence of such narratives (as expressed through individual preferences) are shaped by an individual’s prior identity or conception of what it means to be American. In other words, crafting the narrative through the lens of a cultural-assimilative American identity most likely affords disliking or silencing critical versions of that narrative. Finally, regardless of individual preferences, engagement with privileged narratives may serve dominant group ends (e.g., decreased support for inclusive policies). In this way, the results illuminate the structural foundations of privilege and oppression.

### Conflict of Interest Statement

The authors declare that the research was conducted in the absence of any commercial or financial relationships that could be construed as a potential conflict of interest.

## Footnotes

1 Participants were instructed to take and share approximately 10 photos with the researchers. Participants submitted between 3 and 28 photos.

2 Five participants in the “before” condition, and 3 participants in the “after” condition failed to complete the measure on political ideology. All of these participants were non U.S. residents. Moreover, even amongst those who did complete the measures, several participants expressed difficulty in completing the measure. Unfortunately, the experimenters did not record the number of participants who verbally expressed their difficulty. Prior research demonstrates a cultural difference in patterns of relations between political orientation and values (e.g., Thorisdottir et al., 2007) and proposes a multilevel analysis of political orientation (e.g., Haidt et al., 2009). This line of work is consonant with our own observations and suggests that participants’ conceptions of liberalism and conservatism may differ based on their national origin (e.g., U.S. vs. Europe). Future research can further explore the extent to which there are differences in the meaning of these ideological constructs, and whether these differences are associated with immigration-relevant attitudes.

3 Eleven participants failed to fully complete this measure so we analyzed the scores of the remaining 24 participants.

4 Six participants failed to fully complete this measure so we analyzed the scores of the remaining 29 participants.

5 Three participants failed to complete the measures so the analyses were run on the remaining 254 participants.

6 As noted in the introductory section, assimilationist conceptions of identity are associated with ethnocentrism, that is, negative evaluations of immigrants and citizens who do not conform to Anglicized standards (e.g., those of Mexican origin) compared to immigrants and citizens who do conform (e.g., those of Canadian origin).
